# The effect of a maternal mentoring program on the timing of first antenatal care visit among pregnant women in Bantul, Indonesia: Results of a cluster randomized trial

**DOI:** 10.34172/hpp.2021.39

**Published:** 2021-08-18

**Authors:** Yhona Paratmanitya, Siti Helmyati, Detty Siti Nurdiati, Emma C. Lewis, Joel Gittelsohn, Hamam Hadi

**Affiliations:** ^1^Department of Nutrition, Faculty of Health Sciences, the University of Alma Ata, Indonesia; ^2^Faculty of Medicine, Public Health, and Nursing, Universitas Gadjah Mada, Indonesia; ^3^Center for Health and Human Nutrition, Faculty of Medicine, Public Health, and Nursing, Universitas Gadjah Mada, Indonesia; ^4^Doctorate Study Program, Faculty of Medicine, Public Health, and Nursing, Universitas Gadjah Mada, Indonesia; ^5^Department of Nutrition and Health, Faculty of Medicine, Public Health, and Nursing, Universitas Gadjah Mada, Indonesia; ^6^Department of Obstetric & Gynecology, Faculty of Medicine, Public Health, and Nursing, Universitas Gadjah Mada, Indonesia; ^7^Center for Human Nutrition, Bloomberg School of Public Health, the Johns Hopkins University, Baltimore, MD, USA; ^8^Graduate School of Public Health, the University of Alma Ata, Indonesia; ^9^Director of Community-Alma Ata Partnership Through Updated Research and Education (CAPTURE), the University of Alma Ata, Indonesia

**Keywords:** Maternal health, Indonesia, Preconception care, Pregnancy, Prenatal care

## Abstract

**Background:** Antenatal care (ANC) is low in developing countries, with an estimated 20% of Indonesian women not initiating ANC during the first trimester. The present study sought to determine the impact of a mentoring program on the timing of the first ANC visit.

**Methods:** This cluster randomized controlled trial was conducted in 3 subdistricts of the Bantul District, divided into 61 clusters per treatment arm, with a final sample size of 205 confirmed pregnant women. The mentoring program consisted of (1) health education, (2) monitoring, and(3) text-message reminders. The primary outcome was the timing of first ANC visit. A multilevel mixed-effect logistic regression model was used to measure the effect of the program on the likelihood of having an earlier first ANC visit, with statistical significance at α=0.05.

**Results:** At the individual-level, the intervention group had a mean time of first ANC visit±2 days earlier than the control group (P<0.05). After adjusted for cluster and other covariates, the odds of starting the first ANC visit early (<39 days of gestation) was higher in the intervention group (adjusted odds ratio [AOR] 3.00; 95% confidence interval [CI] 1.17-7.72).

**Conclusion:** Maternal mentoring can improve the timing of the first ANC visit. This program has the potential to be adopted by health care systems in settings where there is little education on the importance of ANC. Future research could extend the length of mentorship until delivery in order to better understand the relationship between mentorship and early ANC on pregnancy outcomes.

## Introduction

The maternal mortality rate (MMR) in Indonesia has decreased over the last three decades but still remains relatively high (305 per 100 000 live births).^1^ Serious efforts are required to reduce MMR in order to reach the Sustainable Development Goals target by 2030 of <70 per 100 000 live births. One strategy is to ensure every pregnant woman receives quality antenatal care (ANC) services. Previous studies have shown that among women with no ANC, there is increasing risk of maternal mortality,^2,3^ and missed opportunities of institutional delivery.^4^

According to the Indonesian Basic Health Survey (2018), more than 90% of pregnant women have received ANC at least once, but only 80% had their first ANC visit during the first trimester and only 74.1% received ANC as recommended with a frequency of at least 4 times during pregnancy (i.e., once during the first trimester, once during the second trimester, and twice during the third trimester).^5^ In other words, about one-fifth of pregnant women are late in receiving their first ANC visit, and one-quarter of pregnant women do not achieve the recommended frequency and timing of ANC visits during pregnancy. Given the high number of pregnant Indonesian women in the last 3 years of more than 5.2 million per year,^6–8^ the lack of ANC in this setting should be considered an important public health challenge.

Previous studies have shown that having an earlier ANC visit during pregnancy is associated with improved pregnancy outcomes, such as reduced risk of (1) gestational hypertension and spontaneous abortion,^9^ (2) low birth weight,^10^ (3) preterm birth^11^, as well as (4) greater chance of consuming 90 tablets or more of iron supplements, in line with recommendations.^12^ Likewise, delaying the first ANC visit can impede the delivery of crucial information about recommended health behaviors. One study in Uganda found that among 400 pregnant women who were late for their first ANC visit (first visit at >20 weeks of gestation), most women did not know (1) at what gestational age they should have their first ANC visit, and (2) the importance of receiving ANC early on. This study recommended that women be educated on ANC, even before pregnancy.^13^

Delivering health education is one strategy for improving health-related behaviors. Our previous study showed that nutrition education followed by short messaging reminders improved knowledge and maternal behavior, improved compliance to iron pill supplementation, and increased hemoglobin levels of pregnant women.^14^ Other studies also found that preconception health counseling and education can increase maternal knowledge, self-efficacy, improved ANC attendance, and maternal lifestyle choices during pregnancy to prevent unwanted pregnancy outcomes.^15,16^ The present study integrated health education into a maternal mentoring program from preconception until pregnancy. The educational materials highlighted (1) the importance of preconception health, (2) recommendations for receiving regular and timely ANC, and (3) education regarding following a healthy diet. Messaging was used as a strategy for reminding women to schedule ANC visits as soon as they experience pregnancy signs and symptoms. The overall goal of the present study was to determine the effect of our adapted maternal mentoring program on the timing of the first ANC visit among pregnant Indonesian women.

## Material and Methods

### Design and setting

The present study was a cluster randomized trial using the hamlet, or small village, as the unit of randomization. The study began in January 2019 and ended in April 2020, and was conducted in three sub-districts of Bantul district, Yogyakarta, Indonesia, with a lower prevalence of ANC and a higher prevalence of anemia during pregnancy compared to the other sub-districts based on data from the Bantul District Health Office.^17,18^ Together, these three sub-districts comprise of 12 villages and 122 hamlets. These 122 hamlets were considered clusters for the purpose of this study, and were divided randomly using random number generator into the intervention group (n = 61) and the control group (n = 61). All eligible pregnant women in each cluster were then followed up with until delivery.

### Participants

**Figure 1:**
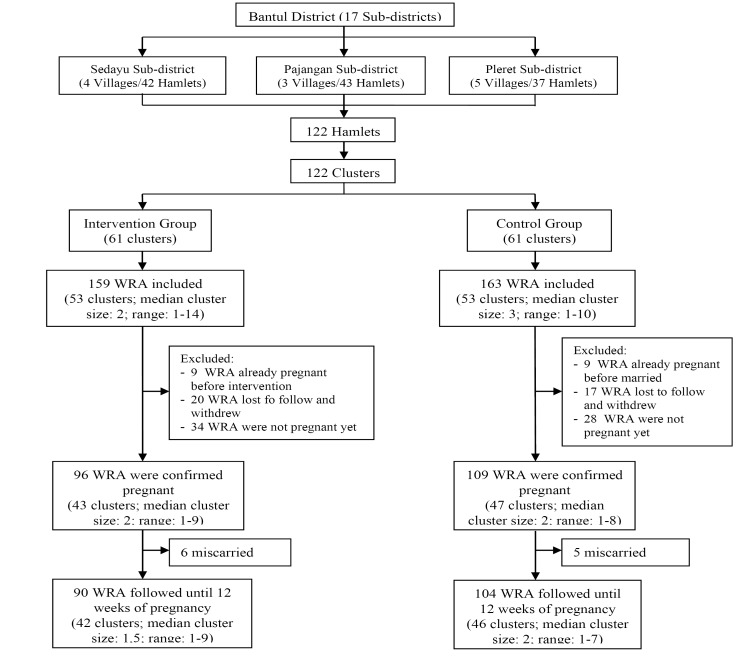
Procedures for selection of the study sample

Due to the cluster design of the study, eligibility criteria were applied at both the individual- and cluster-level. At the cluster-level, all hamlets located in Sedayu, Pajangan, and Pleret sub-districts were included. Hamlets with no eligible samples were excluded from the analysis. At the individual-level, those included were (1) women of reproductive age planning for pregnancy, who (2) planned to stay in the research area for at least the next 2 years, and (3) were willing to take part in the research by signing the informed consent. Women who were already pregnant before the mentoring program began, or who were lost to follow up, were excluded from the final analysis. At the individual-level, the present study included 322 preconception women at baseline, of which 205 were later confirmed to be pregnant and were included in the outcome measure of timing of the first ANC visit (Figure 1). Confirmation of pregnancy status was carried out in several stages: (1) asking the respondents once a month via WhatsApp (WA)/short message service (SMS) whether they had signs of pregnancy or not during the last month; (2) if not, then they were asked again in the following month, but if so, then the next question was whether it had been confirmed by the midwife/doctor or not; and (3) if not, then they were asked to immediately see a midwife/doctor, but if so, it meant that there was a confirmed pregnancy.

### Interventions 

The intervention group received maternal mentoring from preconception until 12 weeks pregnant, whereas the control group received usual routine health services. The maternal mentoring program was integrated into a public health surveillance activity under CAPTURE (Community-Alma Ata Partnership Through Updated Research and Education), a collaboration between the University of Alma Ata, the Bantul District Health Office, and the Bantul Regency Government. In the present study, the maternal mentoring program included (1) preconception health education, which was provided once during the first home visit, delivered by face-to-face counseling with a booklet as the education media, (2) monitoring of pregnancy status by asking this question via WA/SMS once a month: “have you experienced signs and symptoms of pregnancy such as late menstruation, nausea, vomiting, or others?”, and (3) after respondents experienced signs and symptoms of pregnancy, they were sent a reminding message to book their first ANC visit immediately. Unlike in the control group, women in the intervention group received maternal education as well as monitoring of their pregnancy status, and reminders to be timely with visiting primary health centers or health professionals, and to comply with recommended iron supplementation. An example of a message sent to women in the intervention group is as follows: “Hi, good morning ma’am. Don’t forget to check your pregnancy at the nearest health center. The earlier the better”. The mentors were trained faculty and students at the University of Alma Ata. Mentors provided the intervention and acted as data collectors. The booklet used as an educational media in this study was developed by the research team through several stages, including a literature review and focus group discussions with health workers and expert panels. The booklet contained: (1) the definition of the preconception period, (2) the importance of paying attention to health during the preconception period, (3) what should be done to prepare for a healthy pregnancy (“The Four Pillars to a Healthy Pregnancy”), and (4) the signs and symptoms of pregnancy. Currently in Indonesia there is no standardized educational media for preconception health and nutrition.

### Outcomes 

We evaluated the effect of a maternal mentoring program begun during the preconception period on the timing of the first ANC visit among pregnant Indonesian women. The primary outcome was the mean time it took to have the first ANC visit after becoming pregnant, and secondary outcomes included change in level of preconception health knowledge, and anthropometric changes (weight, height, mid-upper arm circumference [MUAC]), pre- and post-intervention.

Data taken at baseline included anthropometric data (weight, height, MUAC) and level of preconception health knowledge, and were measured during the preconception period in the first home visit before being given the counseling. The time at which a pregnant woman had her first ANC visit was expressed in terms of the mother’s gestational age in ‘days’. The gestational age was obtained from the difference between the date of the first ANC visit and the mother’s first day of last menstruation. The date of the first ANC was obtained from the records in the mother’s Maternal and Child Health Handbook. At the end of study, mentors made a second and final home visit to measure weight, MUAC, and level of knowledge about preconception health.

### Sample size calculation

We calculated sample size using a method that takes into account the design effect (DE) of clustering. We assumed an intra-cluster correlation of 0.05 and the average cluster size was 2.64, resulting in a DE of 1,082. We use a power of 80% to detect a 10% difference in the proportion of first time ANC visits between the two groups, resulting in a minimum sample size of 112 respondents per group.

### Data collection

Basic characteristics and anthropometric measurements (height, weight, and MUAC) were collected on the basis of a standardized protocol by well-trained interviewers. The standing height was measured by a multi-function brand Stadiometer with a capacity of 2 m and a precision of 0.1 cm. Body weight was measured by a Camry digital weight scale with a capacity of 150 kg. The weight scale was calibrated daily before using it. The body mass index (BMI) was calculated as weight (kg)/height squared (m^2^) and was determined on the basis of the World Health Organization (WHO) criteria for the Asian population: normal weight (18.5 to <23 kg/m^2^), overweight (23.0 to <27.5 kg/m^2^), and obese (≥27.5 kg/m^2^). MUAC was measured using standard methodology.^19^ We used a MUAC cut-off value of <23.5 cm to define malnourished pre-conception women. Data on socio-demographic were collected by well-trained interviewers by using structured questionnaires. The level of preconception health knowledge was measured by well-trained interviewers using structured questionnaires consisting of 25 questions at the first home visit, before being given the counseling. The score of preconception health knowledge ranged from 0 to 100, with the mean score 57.9 (±11.8). We got a value of internal consistency Cronbach’s of 0.7 indicating that the questionnaire is reliable. All interviewers were bachelor students in the school of nutrition, Faculty of Health Sciences, the University of Alma Ata.

### Statistical analysis

For categorical data, we used frequency distributions to present the data. For continuous data, we used mean and standard deviation (SD) for normally distributed data, and median for non-normal data. Pearson’s chi-squared test (for categorical variables) and independent t- test or Mann-Whitney test (for continuous variables) were used to test the difference between intervention and control groups across different characteristics at baseline. Kolmogorov-Smirnov was used to test the data normality. To examine the effect of the mentoring program on the likelihood for earlier first ANC visit, we defined early first ANC visit as the ANC visit before the 39^th^ day of gestational age or not early first ANC visit if the ANC visit was done after the 39^th^ day of gestational age. The cut-off of 39^th^ day was the median of the first ANC visit in this study. Using a multilevel mixed-effect logistic regression model, we measured the effect of the mentoring program on the likelihood of having earlier first ANC visit adjusting for cluster and other covariates. This analysis was conducted to account for within cluster variation as well as to adjust for other potential confounders.^20^ Furthermore, to examine the effect of mentoring program on the increment of knowledge score, weight, and MUAC before pregnancy and after 13-16 weeks of gestation at individual level, we performed a multilevel mixed-effect linear regression adjusting for cluster and other covariates.^21^ We performed all data analyses using STATA v.15 MP (StataCorp LLC, Texas, USA).

## Results

Figure 1 displays the participant flow. From a total of 122 clusters, there were 16 clusters (8 clusters in each group) that unavailable because no preconception women met the criteria. At baseline, 322 preconception women were eligible to participate (159 women in the intervention groups and 163 women in the control groups). At endline, 205 preconception women were available for final measurement. One hundred and seventeen participants were unavailable because of lost to follow, not pregnant until the end of the study, or already pregnant before the intervention or before marriage.

The baseline characteristics of the sample are shown in Table 1. There was no significant difference found between intervention and control groups for all baseline characteristics. The majority of the respondents were between 20 to 35 years of age, and about 61.5% of the respondents had a middle-school education. 73.7% of the respondents were currently working, and the majority (60.3%) earned less than the district minimum monthly wage. The mean monthly income in the intervention group was slightly lower than in the control group, but this difference was not statistically significant. Regarding nutritional status, the percent overweight in the intervention group was slightly higher than the percent underweight, but in the control group the percent overweight was lower than the percent underweight. 36.5% of the respondents in the intervention group and 34.9% in the control group were deemed to be at risk of chronic energy deficiency with a MUAC of less than 23.5cm.

**Table 1:** Baseline characteristics of the study participants

**Variable**	**Intervention (n = 96)**	**Control (n = 109)**	***P*** ** value**
Age (y), mean ±SD	25.15 ± 4.45	24.13 ± 3.50	0.120
Age groups, %			0.626
<20 and >35	7/96 (7.3)	10/109 (9.2)	
20-35	89/96 (92.7)	99/109 (90.8)	
Education, %			0.183
≤9 years	5/96 (5.2)	13/109 (11.9)	
10-12 years	59/96 (61.5)	67/109 (61.5)	
>12 years	32/96 (33.3)	29/109 (26.6)	
Working status, %			0.927
Working	71/96 (74.0)	80/109 (73.4)	
Not working	25/96 (26.0)	29/109 (26.6)	
Monthly income (rupiahs)^a^, mean ± SD	1 612 676 ± 693 475	1 688 562 ± 668 508	0.392
Income level^a^, %			0.161
<District minimum monthly wage	47/71 (66.2)	44/80 (55.0)	
≥District minimum monthly wage	24/71 (33.8)	36/80 (45.0)	
BMI (kg/m^2^), mean ± SD	22.39 ±4.70	21.65 ±4.92	0.169^b^
Preconception nutritional status, %			0.563
Underweight (BMI <18.5)	14/96 (14.6)	22/109 (20.2)	
Normal (BMI 18.5-25.0)	67/96 (69.8)	70/109 (64.2)	
Overweight/obese (BMI>25.0)	15/96 (15.6)	17/109 (15.6)	
MUAC (cm), mean ± SD	25.30 ±3.90	24.96 ±3.38	0.850^b^
At risk of chronic energy deficiency, %			0.812
Yes	35/96 (36.5)	38/109 (34.9)	
No	61/96 (63.5)	71/109 (65.1)	

Abbreviations: SD, standard deviation; BMI, body mass index; MUAC, mid-upper arm circumference. ^a^Minimum monthly wage of Bantul District in 2019 was IDR 1.649.800 (±115 USD), based on the governor's decree. ^b^ Analyzed using Mann-Whitney test.

Table 2 demonstrates that at the individual-level, the mean time for the first ANC visit in the intervention group was significantly different from the control group (*P* < 0.05), while at the cluster-level, the difference was not statistically significant (*P* > 0.05), though did trend towards significance. Pregnant women in the intervention group made their first ANC visit ±2 days earlier than pregnant women in the control group. In the intervention group, the average of their first ANC visit was at 39.75 (±7.71) days of gestation. Meanwhile, in the control group, the average first ANC visit was at 41.96 (±9.72) days. The gestational age range of the first ANC visit timing was 24-92 days of pregnancy. Further analysis was conducted at individual level adjusting for cluster to account for within cluster variation.

**Table 2:** Mean difference of first ANC visit timing between intervention and control groups

**Variable**	**Individual level analysis**		***P*** **value** ^a^	**Cluster level analysis**		***P*** **value** ^a^
	**Intervention** **(n = 96)**	**Control** **(n = 109)**		**Intervention** **(n = 42)**	**Control** **(n = 46)**	
Mean of first ANC visit timing (gestational age in days)	39.75 ±7.71	41.96 ±9.72	0.020*	39.85 ±7.55	41.77 ±7.35	0.073
∆ Time of first ANC visit	2.21			1.92		

Abbreviation: ANC, antenatal care. ^a^Using Mann-Whitney test because the data is not normally distributed. *P < 0.05

Bivariate analysis was carried out to examine determinants of first ANC visit timing using a simple logistic regression model as well as a simple multilevel mixed-effect logistic regression model adjusting for cluster (Table 3). Based on cluster adjusted analysis, respondents aged 20-35 years, with a duration of education more than 9 years, having income level above the district minimum monthly wage, having a normal BMI, and at risk of chronic energy deficiency, have a greater chance to make their first ANC visit <39 days of gestation, but none of these associations were statistically significant (Table 3).

**Table 3:** Determinants of the first ANC visit timing

**Variable**	**Time of 1** ^st^ ** ANC visit**		**Crude OR (95% CI)** ^b^	**Cluster adjusted OR (95% CI)** ^c^
	**<39 days** ^a^	**≥39 days** ^a^		
Treatment group				
Intervention (received mentoring program)	52 (55.9)	44 (39.3)	1.96 (1.12-3.43)	2.00 (1.06-3.80)
Control	41 (44.1)	68 (60.7)	1	1
Age (years), %				
<20 and >35	6 (6.5)	11 (9.8)	1	1
20-35	87 (93.5)	101 (90.2)	1.58 (0.56-4.45)	1.66 (0.53-5.16)
Education, %				
≤9 years	5 (5.4)	13 (11.6)	1	1
10-12 years	60 (64.5)	66 (58.9)	2.36 (0.80-7.02)	2.76 (0.80-9.54)
>12 years	28 (30.1)	33 (29.5)	2.21 (0.70-6.95)	2.35 (0.65-8.49)
Working status, %				
Working	69 (74.2)	82 (73.2)	1	1
Not working	24 (25.8)	30 (26.8)	0.95 (0.51-1.78)	1.01 (0.51-2.03)
Income level^a^, %				
<District minimum monthly wage	42 (60.9)	49 (59.8)	1	1
≥District minimum monthly wage	27 (39.1)	33 (40.2)	0.95 (0.50-1.83)	1.06 (0.48-2.35)
Nutritional status (BMI), %				
Underweight	17 (18.3)	19 (17.1)	0.95 (0.45-1.98)	0.98 (0.43-2.22)
Normal	66 (71.0)	70 (63.1)	1	1
Overweight	10 (10.8)	22 (19.8)	0.48 (0.21-1.09)	0.46 (0.19-1.13)
At risk of chronic energy deficiency, %				
Yes	37 (39.8)	36 (32.1)	1.39 (0.79-2.48)	1.52 (0.79-2.94)
No	56 (60.2)	>76 (67.9)	1	1

Abbreviations: ANC, antenatal care; OR, odd ratio; CI, confidence interval; BMI, body mass index. ^a^Median value; ^b^ Analyzed using a simple logistic regression model; ^c^ Analyzed using a simple multilevel mixed-effects logistic regression model adjusting for cluster (n cluster = 88)

**Figure 2:**
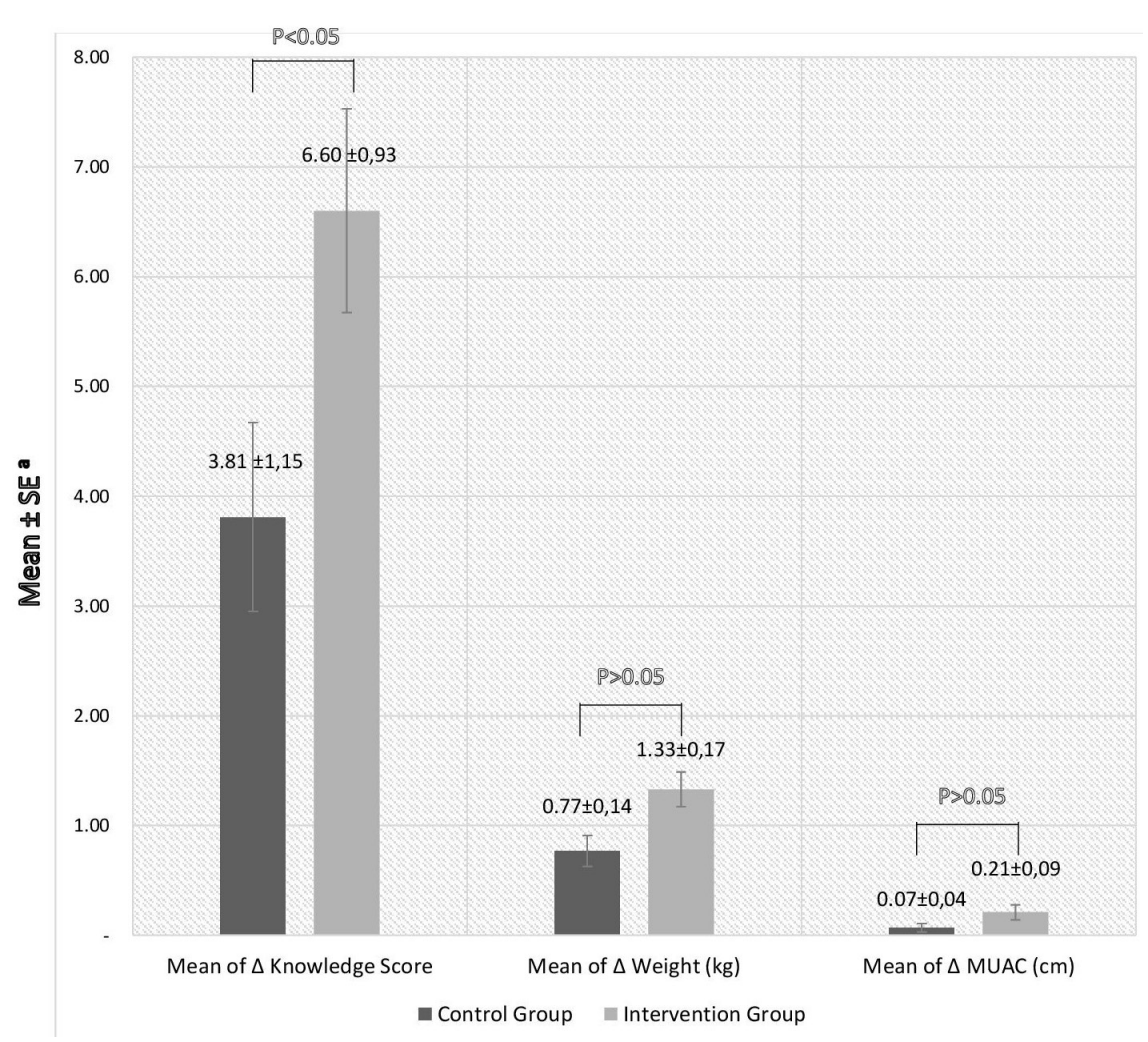
The increment of knowledge score, weight, and MUAC by treatment group
^a^Mean increment and SEs were generated from a multilevel mixed-effect linear regression model adjusting for cluster and other covariates”

In multivariate analysis, after adjusting for cluster, we found that respondents who received the mentoring program were 3 times more likely to make ANC visits earlier, at gestational age <39 days, than those in the control group (OR, 3.00; 95% CI, 1.17-7.72) adjusting for age, education level, income level, and preconception nutritional status (Table 4). The effect of mentoring program on the increment of knowledge score, body weight and MUAC was shown in Figure 2. Increment was determined by analyzing the difference between pre-pregnancy (preconception) and 13-16 weeks’ gestation. Women in the intervention groups had a significantly greater increment in knowledge score than the control groups. They also had a higher increment in body weight (72.7% higher) and in MUAC (more than double), but the difference was not statistically significant (Figure 2).

**Table 4:** Effect of mentoring program on the timing of the first ANC

		**Adjusted OR** ^a^ ** (95% CI)**	**Cluster Adjusted OR** ^b^ ** (95% CI)**
Treatment	Intervention (receiving mentoring program)	2.58 (1.30-5.11)	3.00 (1.17-7.72)
	Control	1	1

^a^Adjusted OR was generated from a multiple logistic regression, adjusting for age, education level, income level, and nutritional status. ^b^Cluster adjusted OR was generated from a multilevel mixed-effect logistic regression, adjusting age, education level, income level, and nutritional status.

## Discussion

This is one of the first intervention trials to be conducted among Indonesian women with the aim of improving prenatal care. Our findings indicate that women in the group that received the mentoring program, on average, made their first ANC visit 2 days earlier than those in the control group, at both the individual- and cluster-levels. Likewise, women in the treatment group were 3 times more likely to make their first ANC visit earlier (<39 days of gestation) compared to the control group. Previous studies have found similar results, with one study in particular conducted in the United States that found that women who received preconception counselling were 2.05 times more likely to have an ANC visit in the first trimester compared to the group who received no counselling.^22^ Another study in Zanzibar also found that women who received a mobile phone preconception counselling intervention had more than double the odds for attending 4 or more ANC visits.^23^

The present study found that the mean time of the first ANC visit in both treatment groups was within the first trimester (<13 weeks), which is in accordance with the recommendation of the Indonesian Ministry of Health. We can conclude that the level of awareness of the importance of attending ANC visits early on during pregnancy is quite good in the chosen study area. Other studies have found different results, one of which was conducted in Nepal and found that almost half (45%) of the respondents did not have their first ANC visit until >3 months of gestation and 28% did not receive ANC at all.^24^ Similarly, in Zanzibar, one study found that the average time for pregnant women to receive ANC for the first time was at 20 weeks of gestation.^23^ This difference in results is thought to be due to cultural factors that can influence the behavior of mothers in utilizing health services. Some previous studies have shown that maternal health care utilization can be influenced by culture.^25,26^

Pregnancy checks are recommended as early as possible, especially because pregnant women who receive ANC since the first trimester have been shown to have a greater chance of having a higher number of ANC visits during their pregnancy. In turn, having a higher number of ANC visits (>4 times) can reduce the risk of experiencing negative birth outcomes such as low birth weight and preterm birth.^27^ An observational study using the secondary data of the 5th wave Indonesian Family Life Survey (IFLS) on 2014 showed that the ANC frequency of short stature mother had a significant relationship with stunting.^28^

First trimester is a crucial period of pregnancy because not only are all of the major body organs and systems of the fetus forming, but also the risk of having a miscarriage is highest during this period. In the intervention group, on average, respondents made their first ANC visit 2 days earlier than in the control group (*P* < 0.05). This difference in time may seem small, but in practice it can also be meaningful when the ANC visit is early, high-risk pregnant women can be detected earlier too. Early detection of symptoms and danger signs during pregnancy is the best effort to prevent serious pregnancy disorder and ensure a safe and healthy pregnancy. In conditions where the mother experiences bleeding, hyperemesis gravidarum, or stomach cramps, it must be immediately checked. If there is a delay in checking for 2 days of course it can endanger the health of the mother and the fetus.

Analyses of secondary outcomes found that the respondents’ knowledge scores increased, both in the intervention and control groups. Although the control group did not receive the educational intervention component, it is possible they increased their preconception health knowledge due to easy access to information using modern day technology. In addition, women entering pregnancy, especially a first pregnancy, tend to improve their health behaviors, such as information seeking.^29^ Not surprisingly, the mean of Δ knowledge score in the intervention group had greater increase than in the control group. This demonstrates that the educational component of our mentorship intervention had a positive impact on respondents’ preconception health and nutrition knowledge. Similar results were found in a study involving pre-marital women in Bandung, where providing education on preconception health significantly increased respondent’s knowledge.^30^

Regarding changes in body weight and MUAC, increases were greater in the intervention group than in the control group. Although statistically not significant, the increases were practically significant. Weight gain during pregnancy is an important indicator for predicting pregnancy outcomes. Inadequate weight gain during pregnancy can increase the risk of giving birth to children with low birth weight,^31,32^ while excessive weight gain during pregnancy can increase the risk of macrosomia.^33^ In the present study, the mean of first trimester weight gain was 1.3 kg in the intervention group and 0.8kg in the control group. Based on the Institute of Medicine Guidelines,^34^ weight gain of 0.5-2 kg in the first trimester is in the normal range. Mid-upper arm circumference is also an important predictor of pregnancy outcomes. Several studies have found that not having an adequate maternal MUAC is associated with low birth weight, preterm birth, and small for gestational age.^35-37^ Energy intake and food availability are some of the factors that can affect maternal MUAC.^38^

The present study has some limitation. The final sample size obtained in the present study did not meet the minimum sample size requirement due to limited time and the fact that less women became pregnant than we anticipated from our initial sample. This limitation resulted in a power decrease to about 77%. This may have led to the absence of a significant difference in the mean time to first ANC visits between the intervention and control groups at the cluster level. Another limitation of this study is related to the intensity of the mentoring program, which may be low. Preconception education maybe should not only be done once at the beginning, but can be strengthened by providing educational messages via WA/SMS on a regular basis. Therefore, further research is needed with an adequate sample size and more intensive interventions.

## Conclusion

In summary, our maternal mentoring program had a significant impact on the time of the first scheduled ANC visit. In addition, respondents who received the program increased their preconception health knowledge. Finally, the increases seen in body weight and MUAC were significantly greater in the intervention group compared to the control group.

This mentorship model can be further developed as a means for (1) monitoring the health of reproductive age women and (2) preparing for a healthy pregnancy. Future research should be done to (1) explore the impact of extending the length of mentorship, and (2) follow women through delivery in order to better understand the relationship between mentorship and early ANC on pregnancy outcomes in this population. Intervention programs like the one implemented in the present study are crucial for reaching the Sustainable Development Goals MMR target for 2030.

## Ethical approval

Ethical clearance was approved by the Medical and Health Research Ethics Committee (MHREC), Faculty of Medicine, Public Health, and Nursing, Universitas Gadjah Mada, Yogyakarta, Indonesia, No. KE/FK/1289/EC/2018 and No. KE/FK/1456/EC/2019. Prior to enrollment, we explain the method of the study to the respondents, and written informed consent was obtained from them. The study was registered at isrctn.com as ISRCTN14448533.

## Acknowledgments

The authors would like to thank all of the women who agreed to participate voluntarily in this study, as well as the research team who collected the data. Furthermore, the support of CAPTURE (Community – Alma Ata Partnership Through Updated Research and Education) of Alma Ata University, Yogyakarta, Indonesia, is greatly acknowledged.

## Funding

Indonesia Endowment Fund for Education, Indonesia Ministry of Finance and the University of Alma Ata.

## Competing interests

None.

## Authors’ contributions

YP, SH, DSN, and HH conceptualized the study. Data collection was carried out by YP. YP, SH, DSN, and HH analyzed the data. The first draft was prepared by YP with subsequent reviews and revisions completed by all authors. All authors reviewed the final draft and gave approval to publish. All authors agreed to be responsible for all aspects of the work in ensuring that questions related to the accuracy or integrity of any part of the work are appropriately investigated and resolved.
